# A Study on Hospitalized Patients’ Payment in South of Iran after the First Round of Health Sector Reform

**Published:** 2017-02

**Authors:** Kamran BAGHERI LANKARANI, Sulmaz GHAHRAMANI, Behnam HONARVAR

**Affiliations:** Health Policy Research Center, Institute of Health, Shiraz University of Medical Sciences, Shiraz, Iran

## Dear Editor-in-chief

Health system reforms are implemented for improvement of the effectiveness, efficiency, quality, equity of health systems and for the ultimate purpose of universal health coverage (1). There is a wide range of experiences with the implementation of health sector change in different countries in the last decade.

In Iran, recently Ministry of Health and Medical Education announced master plan for health system reform (HSR) (2). Accordingly, the ministry intended to implement set of seven instructions (3). The first and most focused of these instructions’ plan is reduced patients’ payments (PP) for inpatient medical services in faculty hospitals with the goal of patient protection against high medical cost. Copayment for patients covered by basic health insurance was fixed to 10% in faculty hospitals and these hospitals were obligated not to refer the patients for purchase of any medicines, equipment, and diagnostic services out of hospital. However, critics have challenged whether this plan could be considered as a major reform as most of the emphasis till now was on inpatients services in governmental hospitals with little attention to other aspects of health system specially outpatient care (which includes high proportion of family payments for health care) (4) and preventive and health promoting plans at national level.

This cross-sectional study purposed to evaluate PP before and after (Oct 2013–Oct 2014) implementation of the first instruction of HSR in one of major hospitals of the country in Shiraz, Iran through a matched (according to sex, age, and duration of admission and diagnosis of patients) comparative study.

Extracted discharge bill from the hospital information system showed that mean percent of PP/total bill ± SD (Median) for hospital services for patients who had a health insurance was 0.15± 0.20 (0.10) after implementation of reform (vs. 0.27± 0.6 (0.16) before reform), this change was statistically significant (*P*<0.001). Because gain in this goal is borderline, therefore, achievement of goal of 10% PP payment through this reform needs more planned efforts. However, new NHA studies are strongly recommended to explore the real trend of PP payments in Iran. PP payment for inpatient and rehabilitation services was 6.5% in last Iran’ National health account 2008 (5).

Mean of PP decreased significantly for cost of medical equipment used inward in the line with the goal of decrease in PP in health sector reform, but mean of total bill for medical equipment used inward increased significantly after implementation of health reform. Surprisingly, there is higher mean value of total bill in Oct 2014 and for laboratory test, mean of total bill and mean of PP both increased, (however, this was not statistically significant) and in *medication cost*, both mean of total bill (not statistically significant) and mean of PP (statistically significant) were increased after reform (Table 1). Since this health system reform has covered only cost of governmental hospitals, the trend toward more hospital stay, more use of drugs and facilities and in more general, inducing demand should be cautioned. The difference in mean of PP for out of hospital services, asked from discharged patients through phone call was significantly decreased (*P*= 0.035) after health sector reform and it is a successful indicator for performance of this reform, however, probability of recall bias should be considered and further longitudinal studies are suggested.

**Table 1:** Mean of total bill and PP for medication, ward requested laboratory test cost, medical equipment had used in ward (IUSD)

**Variable**		**October 2013**	**October 2014**	***P*-value**
Medication	Mean of total bill ± DS (Median)	163.77 ± 232.28 (78.41)	189.76± 277.73 (79.52)	0.99
	Mean of PP±DS (Median)	18.17 ± 7.60 (10.51)	32.52± 109.84 (4.88)	0.018
Laboratory test cost	Mean of total bill ± DS (Median)	163.77 ± 232.28 (78.41)	189.76± 277.73 (79.52)	0.76
	Mean of PP±DS (Median)	18.74 ± 27.60 (10.51)	32.52± 109.84 (4.88)	0.13
Medical equipment used in ward	Mean of total bill ± DS (Median)	78.11 ± 129.47 (20.85)	164.82± 296.59 (64.16)	0.004
	Mean of PP±DS (Median)	64.09 ± 149.22 (14)	50.36± 135.70 (7.65)	0.006

Comparison of costs between mean of total bill, PP, out of hospital PP in different wards shows that there is no significant change after implementation of reform except in mean of PP in pediatric ward.

Reforms should be precisely evaluated and, besides, the considering the views of stakeholders, including patients, to prevent and treat unwanted consequences (6), and should be based on scientifically available evidence. For instance in NHA findings performed in 2008 showed, the medication and equipment group, was first in household health expenditures ranking, was purchased mostly from drug stores entail 83% of total cost, second one was outpatient services in home, office or clinic and entails dentistry services (59%), medical and paramedical 34% and 7%, respectively (5). Health system reform should consider this highly expensive services if the goal of reform is a permanent decrease in PP.
